# Mining beyond the exome

**DOI:** 10.1186/1756-0381-4-14

**Published:** 2011-06-13

**Authors:** Davnah Urbach, Jason H Moore

**Affiliations:** 1 Dartmouth College, Institute for Quantitative Biomedical Sciences, One Medical Center Dr., Lebanon, NH 03756, USA; 2 Dartmouth Medical School, Department of Genetics, One Medical Center Dr., Lebanon, NH 03756, USA; 3 Dartmouth Medical School, Department of Community and Family Medicine, One Medical Center Dr., Lebanon, NH 03756, USA

## 

In the late 18^th ^century, Erasmus Darwin, Charles Darwin's grandfather, advocated evolutionary theory as a mean to "unravel the theory of disease". More than 200 years later, although Darwinian medicine is regaining some ground after having been muzzled during the second half of the 20^th ^century, genomics has largely outcompeted evolution and has acquired a dictatorial success as a tool for studying disease etiology [1]. From an evolution-inspired perspective, we have gradually drifted into the habit of focusing primarily on genomic data from sources such as genome-wide association studies (GWAS). As a result, understanding the how and why of human diseases and pathobiology has largely become a matter of crunching DNA sequences. Despite the popularity of GWAS, their reality remains unchanged: most of the susceptibility loci they allow to identify explain only a small fraction of the heritability of complex diseases [2]. A number of reasons for the so-called "missing heritability" have been proposed [2], and our goal is not to review them all. Here we primarily reiterate that there is more to discover than non-synonymous point mutations and suggest that amid genetic deserts and genetic islands, there is also more to explore than the coding regions of the genome. We then highlight the importance and the necessity of designing efficient methods to mine beyond the exome.

The premise of GWAS is the "common disease-common variant" hypothesis, which posits that common diseases are, at least partly, associated with DNA sequence variations or polymorphisms present in more than 1-5% of the population. It turns out that most allele frequencies battle to reach the 5% detection threshold of commercial genotyping arrays and the "common disease-rare variant" hypothesis is gradually taking precedence over its counterpart [2]. Hence, aiming for the rare variants using whole genome sequencing for example is one first step into the right direction [3]. A further step is to deliberately include synonymous polymorphisms among the genetic variants considered in association studies. Although largely disregarded, synonymous polymorphisms are about twice as numerous as non-synonymous ones [4] and are often found responsible for altered protein structure, function and expression level [5]. Accordingly, a considerable list of disease-associated synonymous polymorphisms is already available [5] and there are more to be found. Besides single nucleotide polymorphisms (SNPs), variation can also be structural: multi-kilobase genomic regions can be inserted or deleted (copy number variation, CNV), or they can be moved (copy neutral variation), within (inversion) or between (translocation) chromosomes [6,7]. Structural variants have already been shown to contribute to disease phenotypes [8,9], but with the help of high resolution GWAS purposely designed to detect them, there are undoubtedly more discoveries ahead [6,7].

Variants can adopt different forms but they can also occur in different locations throughout the genome. When given the choice between (quasi) random SNPs and SNPs located in coding regions (gene-centric approach), choosing the latter is the safer bet [10]. However, the fact that more than 80% of the risk-associated variants identified so far fall outside of the coding regions suggests that there is a third option, namely the non-coding regions of the genome, including intergenic regions, introns and 3' and 5' untranscribed regions [11]. Non-coding regions harbor plenty of functional DNA, composed essentially of regulatory elements such as enhancers, promoters, insulators and silencer, and of non-coding functional RNA such as micro-RNA (miRNA). As the non-coding regions of the genome have gradually been revealing their secretes, evidence for their etiological importance has accumulated. Accordingly, genetic variation at regulatory elements [12-15] and at miRNA [16-18] has been found to play an important role in various diseases. Both better SNP coverage and whole genome sequencing will allow for a more methodological exploration of the non-coding regions of the genome.

There is more to the genome than we may have believed. Yet novel discoveries heavily rely on the availability of adequate and powerful analytical tools to exploit rich and complex data. In particular, progress in our understanding of the genetic architecture of common diseases requires efficient methods for merging different types of data and exploiting them simultaneously. Recent literature provides promising ideas on how to combine expert knowledge and crude genotyping data. Cowper et al. [14] for example suggest the use of genome-wide regulatory networks as a framework to incorporate biological knowledge to the analysis and interpretation of genotyping data, including data collected in the non-coding regions of the genome. This fits into a broader systems genetics approach to human disease [19]. Data are accumulating at a faster rate than methodological tools do. We suggest that there is room and urgent need for more ideas on how to analyze and integrate the different sources of information that we extract from both popular and remote regions of the genome. The last five years has focused on the task of manipulating large genomic data sets. Now is the time to integrate and synthesize these disparate sources of genomic information.
